# Editorial: Haplotype Analysis Applied to Livestock Genomics

**DOI:** 10.3389/fgene.2021.660478

**Published:** 2021-05-18

**Authors:** Gábor Mészáros, Marco Milanesi, Paolo Ajmone-Marsan, Yuri Tani Utsunomiya

**Affiliations:** ^1^Department of Sustainable Agricultural Systems, University of Natural Resources and Life Sciences, Vienna, Austria; ^2^Department for Innovation in Biological, Agro-food and Forest Systems-DIBAF, Università della Tuscia, Viterbo, Italy; ^3^Department of Production and Animal Health, School of Veterinary Medicine, São Paulo State University (Unesp), São Paulo, Brazil; ^4^Department of Animal Science Food and Nutrition - DIANA, Università Cattolica del Sacro Cuore, Milan, Italy; ^5^International Atomic Energy Agency (IAEA) Collaborating Centre on AnimalGenomics and Bioinformatics, São Paulo, Brazil; ^6^AgroPartners Consulting, São Paulo, Brazil

**Keywords:** haplotypes, genome architecture, phasing, recombination, mutation

The recent availability of dense panels of single nucleotide polymorphism (SNP) markers has permitted a finer investigation of genome architecture, a deeper understanding of biology and evolution, and the implementation of marker-assisted and genomic selection in livestock species. Paradigmatic examples of the use of SNP panels include understanding domestication, population diversity, inbreeding, admixture, demographic trajectories, identification of loci associated with economically important traits, and accurate prediction of breeding values. The common denominator of the vast majority of the research conducted in livestock to date has relied on analytical tools that treat genetic markers as individual and independent variables. We know, however, that genetic inheritance is driven by segments of closely interlinked nucleotides. Thus, utilizing phased multi-marker segments (i.e., haplotypes) holds the potential of improving existing models. This is particularly true in genome-wide association studies (GWAS) and genomic predictions, which are analyses that rely on the concept that information of unobserved causal variants is captured by correlation (linkage disequilibrium—LD) with nearby (observed) markers.

The potential utilization of haplotypes in genetic analysis is highly varied. Haplotypes are used in the imputation process. Imputation is the *in silico* procedure that allows us to expand upon our information on sparse SNP markers produced by existing microarray data up to the whole-genome sequence level without additional genotyping and sequencing.

Since haplotypes may serve as better proxies for causal variants than single SNP markers, the incorporation of haplotype data in genomic predictions seems promising in the absence of information on functional alleles. In extensive conditions, e.g., in the tropics, haplotypes could be used to select favorable combinations of variants in crossbreds and advanced backcross programs to retain those important for adaptation to local environmental conditions as well as those for improved production. Also, the models applied to the characterization of livestock genetic diversity could be re-designed to better estimate relationship and inbreeding, facilitate the investigation of difficult traits, as those involved in adaptation to different production systems and ecosystems, and extend the investigation of genotype-by-environment interaction. Future developments in animal breeding and genetics will be strongly based on the increasing availability of data, both molecular and phenotypic. However, our ability to dissect and understand livestock complex traits is still limited. The use of haplotypes instead of single markers and of more correct inheritance models may contribute to a better understanding of the genetics underlying livestock trait complexity and biology.

The “Haplotype Analysis Applied to Livestock” Research Topic is intended to collect empirical studies and theoretical papers exploring, evaluating, and improving the use of haplotype analysis in livestock. After its conclusion, it managed to collect 12 articles from 89 authors, with subjects ranging from relatively straightforward diversity analyses to complex applications to unravel the genetic architecture of quantitative traits. Data used in the research studies were SNP microarray data, whole-genome sequences, or a combination of both.

Haplotype size is influenced by recombination, and consequently by the level of linkage disequilibrium (LD) existing in a population. In livestock, LD has been largely influenced by human decisions since domestication, as humans have ruled livestock demography and recent selection intensity and direction. The extent of LD in livestock is reviewed by Qanbari, with a focus on cattle and chicken populations. The study provides insights into pair-wise allelic correlations and haplotype structure in the genomes of livestock.

The concept of LD was also utilized in the development of hierarchical clustering methods for haplotype-based genomic predictions by Won et al. Their study showed increased accuracies when haplotypes, rather than single SNPs, were used to predict genomic breeding values. Importantly, the authors found that not all traits benefit from the use of haplotype data equally, and that haplotype size should be optimized on a case-by-case basis. Therefore, their results suggest a need for further improvements in methods for haplotype size selection that can consider both population structure and trait architecture.

Haplotypes are also used to detect selection signatures. An example of their utilization is shown by Aliloo et al., who sought genomic regions influencing milk production and carrying selection signals related to variation in environment, climate, and disease challenges on the African continent. The study focused on highly admixed populations of exotic and local cattle breeds. Finding selection signatures related to tolerance to African animal trypanosomiasis in Sheko cattle was the goal of Mekonnen et al. The identified genomic regions were further investigated to find promising candidate genes and over-represented genomic pathways influencing trypanotolerance. A promising regulator appears to be Caspase protease, which could play a role in the design of future intervention strategies to improve the health of cattle populations.

A wider diversity study by Luzuriaga-Neira et al. described the population structure and the relationships among South American chicken populations. Understanding the origin and assessing the extent of genetic diversity is pivotal in safeguarding and valuating animal genetic resources. Unfortunately, local chicken populations are often neglected in this respect. Admixture studies revealed the strong influence of commercial populations but also discovered unusual gene flows within the continent.

The correct identification of haplotypes based on reference genomic data is one of the cornerstones of imputation techniques. Butty et al. compared different methods designed to optimize the selection of samples to compose a reference haplotype library supporting routine imputation. In summary, if the reference set is empty, key ancestors and animals carrying common haplotypes should be the first to be included in the library. Identification of the latter can be conducted with the new Highly Segregating Haplotype method presented by the authors. As the reference set grows, rare alleles become more important, in which case newer reference samples should be selected using the Inverse Selection Method. Faux et al. presented a method for automatically matching haplotypes used for imputation, which utilizes extremely randomized trees in a random forests method. The approach holds great potential in improving imputation accuracy, as well as in developing new applications that rely on haplotype matching, such as identification of deleterious haplotypes or prediction of carriers of complex structural variants.

The power of haplotypes in capturing information about unobserved sequence variants has vast applications. For example, Meier et al. analyzed casein variants in German Black Pied cattle, Xu et al. provided insight into the genomic architecture of scrotal hernia in pigs, Oyelami et al. revealed new candidate genes and QTLs for meat quality and disease resistance in pigs, and Zhang et al. investigated hip-height and muscle development in beef cattle. The overarching theme in these studies was the use of haplotypes, instead of single SNPs, to identify relevant regions of the genome to be used in DNA-assisted breeding programs.

From a general perspective, however, the natural hereditary processes and the genetic architecture of economic traits are profoundly complex. This is in contrast with the need for the development of reliable models. The hierarchical modeling technique developed by Selle et al. combined simulation studies and cattle data with the goal to improve estimates of haplotype effects on traits of interest, especially in cases of limited data availability for rare haplotypes.

The papers included in the Research Topic “Haplotype Analysis Applied to Livestock” are examples of how these genomic segments could be utilized in a wide variety of ways. We invite you to browse and read them with the hope that they widen your overview and give you new ideas for future investigations.

## Author Contributions

All authors contributed to the writing and revision of the manuscript to an equal extent.

## Conflict of Interest

By the time this research topic was completed, YTU was a member of the scientific board of AgroPartners Consulting. The remaining authors declare that the research was conducted in the absence of any commercial or financial relationships that could be construed as a potential conflict of interest.

